# Impact of Molecular Testing Using Next-Generation Sequencing in the Clinical Management of Patients with Non-Small Cell Lung Cancer in a Public Healthcare Hospital

**DOI:** 10.3390/cancers15061705

**Published:** 2023-03-10

**Authors:** Javier Simarro, Gema Pérez-Simó, Nuria Mancheño, Emilio Ansotegui, Carlos Francisco Muñoz-Núñez, José Gómez-Codina, Óscar Juan, Sarai Palanca

**Affiliations:** 1 Molecular Biology Unit, Service of Clinical Analysis, Hospital Universitario y Politécnico La Fe, 46026 Valencia, Spain; 2 Clinical and Translational Cancer Research Group, Instituto de Investigación Sanitaria La Fe (IIS La Fe), 46026 Valencia, Spain; 3 Pathology Department, Hospital Universitario y Politécnico La Fe, 46026 Valencia, Spain; 4 Pulmonology Department, Hospital Universitario y Politécnico La Fe, 46026 Valencia, Spain; 5 Radiology Department, Hospital Universitario y Politécnico La Fe, 46026 Valencia, Spain; 6 Medical Oncology Department, Hospital Universitario y Politécnico La Fe, 46026 Valencia, Spain; 7 Biochemistry and Molecular Biology Department, Universidad de Valencia, 46010 Valencia, Spain

**Keywords:** non-small cell lung cancer, molecular diagnosis, translational research, next-generation sequencing, quality management system

## Abstract

**Simple Summary:**

Precision medicine has revolutionized the treatment of advanced non-small cell lung cancer (NSCLC). Due to the discovery of novel predictive biomarkers, an exhaustive molecular characterization of the disease is required for adequate clinical management. In this research, we aim to evaluate the implementation of next-generation sequencing (NGS) in routine diagnostics under a quality management system. In a cohort of 350 patients, NGS studies were able to reveal a distinct molecular profile of the disease according to sex and smoking status, as well as co-occurring and mutually exclusive relationships between molecular alterations. In stage IV patients, targeted therapies were associated with longer progression-free and overall survival. NGS has expanded precision medicine in our center by increasing the percentage of patients with actionable molecular alterations. Our findings consolidate the use of NGS as a molecular diagnostic tool in the clinical routine of a public healthcare hospital.

**Abstract:**

Next-generation sequencing (NGS) is a molecular approach able to provide a comprehensive molecular profile of non-small cell lung cancer (NSCLC). The broad spectrum of biomarker-guided therapies has positioned molecular diagnostic laboratories as a central component of patient clinical management. Here, we show the results of an UNE-EN ISO 15189:2022 NGS-accredited assay in a cohort of 350 patients. *TP53* (51.0%), *KRAS* (26.6%) and *EGFR* (12.9%) were the most frequently mutated genes. Furthermore, we detected co-occurring and mutually exclusive alterations, as well as distinct molecular profiles according to sex and smoking habits. Actionable genetic alterations were significantly more frequent in female patients (80.5%, *p* < 0.001) and in never-smoker patients (87.7%, *p* < 0.001). When NGS was established as the main molecular testing strategy, 36.4% of patients received at least one line of targeted treatment. Among 200 patients with stage IV NSCLC, first-line treatment with targeted therapies was associated with a longer progression-free survival (PFS) (13.4 months (95% CI, 10.2–16.6) (*p* = 0.001)). Similarly, the overall survival (OS) of patients receiving at least one targeted drug was significantly longer (26.2 months (95% CI, 11.8–40.5) (*p* < 0.001)). Our results show that the implementation of NGS in the public healthcare system has provided a broader application of precision medicine.

## 1. Introduction

The understanding of the genomic alterations that lead to cancer cells’ proliferation has consolidated precision medicine as a new therapeutic paradigm in non-small cell lung cancer (NSCLC) [1]. As a consequence, the molecular profiling of NSCLC has become central to clinical management since the discovery of predictive biomarkers and the clinical impact of targeted therapies.

The development of precision medicine has created the requirement of rapid translational research in the field of molecular diagnosis in order to provide an up-to-date assessment of clinically relevant molecular alterations [2,3]. However, specimen adequacy is a significant issue in the molecular diagnosis of NSCLC. Reduced sample sizes of cytology and tissue biopsies, as well as the quality of nucleic acids after fixation and paraffin-embedding processes, may compromise the success of molecular studies [4].

Due to the actual requirements of precision medicine in NSCLC and the intrinsic limitations of molecular diagnosis, high-throughput approaches are needed. Next-generation sequencing (NGS) has rapidly positioned itself as a central technique in molecular diagnostic laboratories because of its ability to simultaneously assess different molecular alterations in a group of relevant genes.

NGS studies constitute a paradigm shift from single-gene approaches involving multiple steps from nucleic acid isolation to report generation. Molecular testing recommendations strongly suggest the incorporation of quality assurance programs in molecular diagnosis laboratories [5]. As in single-gene approaches, the accreditation of NGS testing with UNE-EN ISO 15189 standards requires an exhaustive validation of the technique, ensuring the traceability of samples and reagents and performing internal and external quality controls. Certification demonstrates that laboratories have a quality management system, are technically competent and produce reliable results [6,7].

NGS provides a comprehensive view of the molecular landscape of NSCLC, deciphering tumor heterogeneity and revealing co-occurring or mutually exclusive genetic alterations. The genetic profiling of NSCLC has revealed differences according to clinical–pathological characteristics, such as ethnicity, histology, sex and smoking status [8,9].

The significant number of actionable molecular alterations that define the molecular spectrum of NSCLC has led current clinical guidelines to strongly recommend the use of gene panels for the molecular diagnosis of NSCLC [10,11,12,13]. Apart from approved targeted therapies, NGS studies are recommended in clinical research centers in order to identify patients eligible for clinical trials with novel targeted agents [11].

In this work, we report the integration of NGS studies into a reference public healthcare hospital under the UNE-EN ISO 15189:2022 quality standard. We evaluated the role of NGS studies in providing a comprehensive molecular profile of patients with NSCLC. Moreover, we assessed the contribution of NGS to identifying patients eligible for targeted drug treatment and the influence of this therapeutic approach on patient outcomes.

## 2. Materials and Methods

### 2.1. Patients and Samples

This study recruited 350 patients with NSCLC who were diagnosed between 2015 and May 2020 at the Hospital Universitario y Politécnico La Fe in Valencia (Spain). The epidemiological and clinical–pathological characteristics of all patients are shown in Table 1. Samples containing at least 150 total cells and 10% of tumor content were considered valid for molecular analyses. The present study was approved by the Drug Research Ethics Committee (CEIm) of the IIS La Fe, and written informed consent was obtained from all patients. The study was carried out in accordance with the Declaration of Human Rights and the Conference of Helsinki.

In January 2021, as a consequence of the accreditation of NGS techniques under the UNE-EN ISO 15189:2022 standard [14], the Lung Cancer Committee of our hospital decided to establish NGS as a routine testing strategy for the molecular profiling of NSCLC. During this period, 128 samples were analyzed using NGS. From sample receipt to report generation, a median turnaround time of 10 days (range: 5–25) was achieved.

### 2.2. Nucleic Acid Isolation

Five 5 μm thick formalin-fixed paraffin-embedded (FFPE) sections were selected for genomic DNA isolation using Deparaffinization Solution and the GeneRead DNA FFPE Kit (Qiagen, Hilden, Germany). Following the manufacturer’s recommendations, RNA was extracted from five 10 μm thick FFPE sections using RecoverAll^TM^ Total Nucleic Acid Isolation Kit (ThermoFisher Scientific, Waltham, MA, USA). The extracted nucleic acids were quantified using a Qubit 3.0 fluorometer with DNA HS or RNA HS Assay Kits (ThermoFisher Scientific, Waltham, MA, USA). NGS studies were conducted at the Molecular Biology Unit (Clinical Analysis Department), an ISO 15189-certified laboratory (Entidad Nacional de Acreditación, ENAC, Nº1302/LE2445).

### 2.3. Next-Generation Sequencing Studies

NGS studies were conducted using Oncomine Solid Tumor (OST; ThermoFisher Scientific, Waltham, MA, USA) in 104 samples and Oncomine Focus Assay (OFA; ThermoFisher Scientific, Waltham, MA, USA) in the remaining 246 samples. Briefly, OST allows the detection of point mutations and small insertions/deletions in 22 genes and fusion transcripts involving 4 genes, while OFA was designed to detect point mutations and small insertions/deletions in 35 genes, copy number variations of 19 genes and fusion transcripts of 23 driver genes. Library preparation and clonal amplification were performed using either manual or automated procedures with the Ion Chef^TM^ Instrument (ThermoFisher Scientific, Waltham, MA, USA) following the manufacturer’s protocols. Clonally amplified libraries were sequenced on the Ion PGM^TM^ System or on the Ion GeneStudio^TM^ S5 System (ThermoFisher Scientific, Waltham, MA, USA). Raw data processing and alignment to the human reference genome (hg19) were performed with the Torrent Server (ThermoFisher Scientific, Waltham, MA, USA), and variant calling/annotation was conducted with the Ion Reporter Server (ThermoFisher Scientific, Waltham, MA, USA). Intronic and synonymous variants were excluded. The filter settings included a total read depth of at least 500X, with a variant read depth of at least 20X. Additionally, filtered-in variants were examined using Integrative Genomics Viewer (IGV) software (Broad Institute, Cambridge, MA, USA).

### 2.4. Statistical Analyses

The quantitative variables are summarized by their mean and standard deviation or median and interquartile range, and the categorical variables are summarized by absolute and relative frequencies. The statistical association between qualitative variables was assessed using the chi-square test or Fisher’s exact test. For progression-free survival (PFS) and overall survival (OS) analyses, patients with incomplete clinical data, without radiological progression and/or who were alive at the time of the analyses were censored. All time-to-event outcomes were estimated using the Kaplan–Meier method and compared across groups using log-rank testing (a univariate analysis). The Cox proportional hazards model was used to evaluate the association between predictor variables and survival. Statistical analyses were performed using GraphPad Prism Software version 7.0.2, (San Diego, CA, USA) and SPSS statistics software v.21 (IBM, Armonk, NY, USA). A *p*-value < 0.05 was considered statistically significant. For the statistical analysis of the data, the clinical evidence of the identified variants (approved drugs and clinical trials) has been reviewed as of May 2022.

## 3. Results

### 3.1. Molecular Alterations Detected Using Next-Generation Sequencing

**Figure 1:**
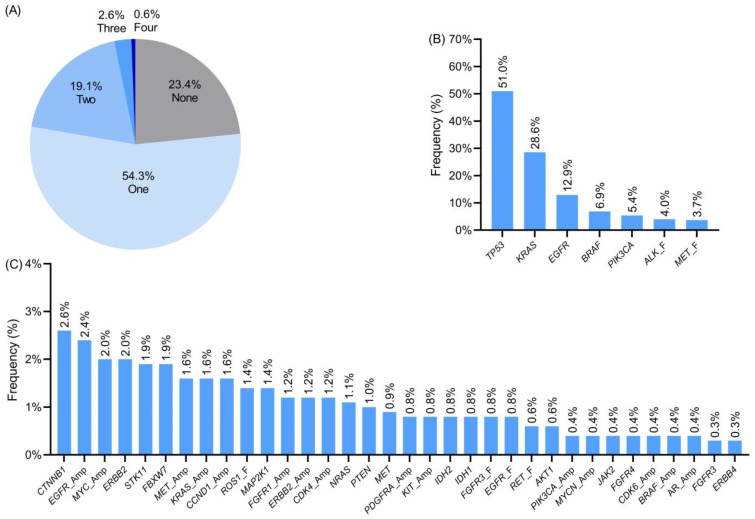
Molecular alterations detected in NGS studies. (**A**) Patient distribution according to the number of genetic alterations detected. (**B**,**C**) Percentage of samples with molecular alterations in the genes included in the study. Variants detected at frequencies of >3% (**A**) and <3% (**B**). Amp: amplification. F: fusion.

In our cohort, 54.3% of patients showed a unique molecular alteration, 22.3% showed at least two molecular alterations (range: 2–4), and 23.4% of patients did not carry any molecular aberration (Figure 1). The most frequently mutated gene was *TP53* (51.0%), followed by *KRAS* (26.6%), *EGFR* (12.9%), *BRAF* (6.9%) and *PIK3CA* (5.4%). Gene fusions involving *ALK* (4.0%), *MET* (3.6%) and *ROS1* (1.4%) were the most common fusion transcripts detected. Regarding copy number variations (CNVs), the gene amplifications of *EGFR* (2.4%) and *MYC* (2.0%) were the most frequently detected (Figure 1).

### 3.2. Clinical–Pathological Associations with Molecular Alterations

**Figure 2:**
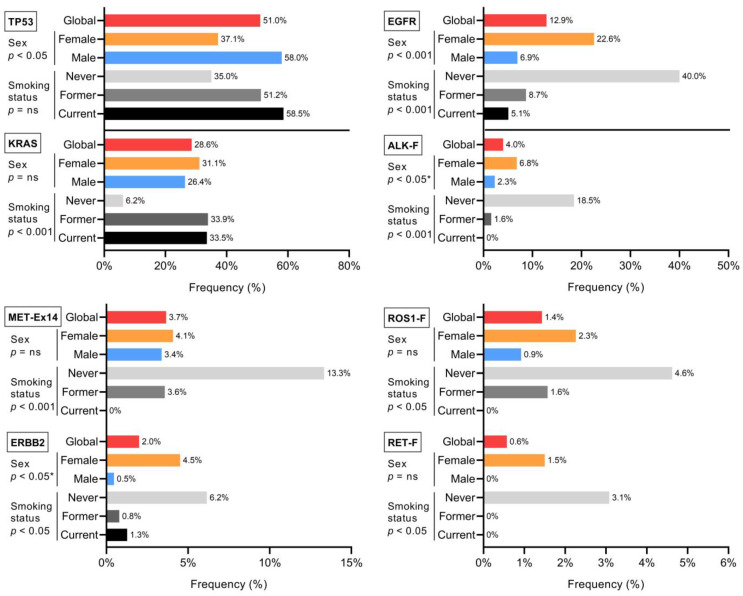
Association of sex and smoking status with specific molecular alterations. Frequency of gene alterations in the global cohort and in the evaluated subgroups is depicted. ns: not statistically significant. * Comparison using Fisher’s exact test.

Regarding molecular alteration associations with clinical–pathological features, smoking status and sex had a strong impact on the mutation profile of the disease. *EGFR* mutations (*p* < 0.001), *ERBB2* mutations (*p* = 0.013) and *ALK* fusions (*p* = 0.049) were more frequently detected in female patients, while *TP53* mutations were significantly associated with male patients (*p* = 0.045). Moreover, *KRAS* mutations were more frequent in former or current smokers (*p* < 0.001), while *EGFR* mutations (*p* < 0.001), *ERBB2* mutations (*p* = 0.029), *ALK* fusions (*p* < 0.001), *ROS1* fusions (*p* = 0.030), *RET* fusions (*p* = 0.012) and METEx14 (*p* < 0.001) were associated with never-smoker patients (Figure 2). Moreover, in former smoker patients, the index of pack/year was significantly lower in the patients harboring *EGFR* mutations (median: 15) than in the patients without *EGFR* mutations (median: 35, U = 302.5, *p* = 0.03) (Figure S1). Regarding age at diagnosis, the patients harboring METEx14 were significantly older (mean 75.1 ± 12.1) than those without this molecular alteration (mean 62.6 ± 11.4), t(244) = −3.2, *p* = 0.002 (Figure S2). No significant differences were observed according to histology.

### 3.3. Co-Occurring or Mutually Exclusive Genetic Alterations

**Figure 3:**
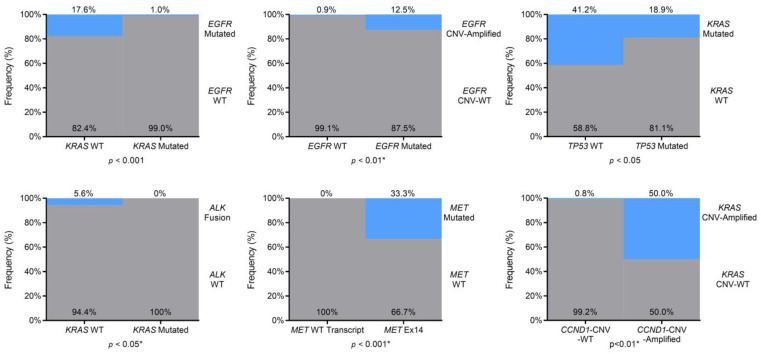
Mosaic plot showing relationships of co-occurrence and mutual exclusivity between the molecular alterations detected in our cohort. For each comparison, the most frequently altered gene is depicted on the *X*-axis. Frequency of patients included in each of the four subgroups defined by the presence/absence of both genetic alterations is depicted in the graph. * Comparison using Fisher’s exact test.

The NGS studies for the molecular profiling of patients with NSCLC revealed co-occurring and mutually exclusive genetic alterations in our cohort. There was a high level of exclusivity between *KRAS* mutations and *EGFR* mutations (*p* < 0.001), *TP53* (*p* = 0.01) and *ALK* fusions (*p* = 0.01). *EGFR* amplifications were more frequent in patients harboring *EGFR* mutations (*p* < 0.01). *MET* mutations were associated with METEx14 skipping (*p* < 0.001), and *KRAS* amplifications and *CCND1* amplifications frequently co-occurred in our patients (*p* = 0.01) (Figure 3). Our analysis suggested the existence of other associations between molecular alterations that did not reach statistical significance: the co-occurrence of *KRAS* mutations + *KRAS* amplifications, *NRAS* mutations + *MYC* amplifications and *CCND1* + *MET* amplifications and a mutual exclusion between *TP53* mutations and *ALK* fusions.

### 3.4. Clinically Relevant Genetic Variants

**Figure 4:**
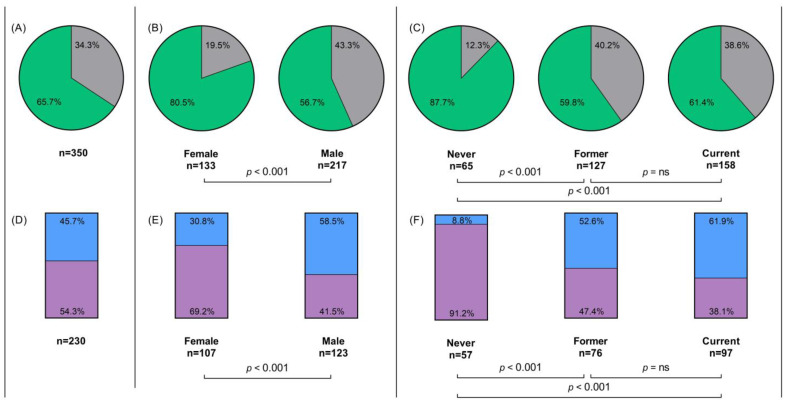
Actionable molecular alterations detected using NGS in our cohort. Percentage of patients with actionable genetic variants (green) in the global cohort (**A**), according to sex (**B**) and according to smoking status (**C**). Patient distribution according to the level of evidence of the detected variants in the global cohort (**D**), according to sex (**E**) and according to smoking status (**F**). Purple: patients who are candidates for treatment with approved drugs, blue: patients eligible for phase I–II clinical trials. ns: not statistically significant.

In our cohort, actionable molecular alterations were detected in 65% of patients. Of these, 54.4% had molecular alterations for which there is an approved targeted therapy, while 45.6% could be enrolled in phase I or phase II clinical trials based on their molecular profile. Actionable molecular alterations were significantly more frequent in female patients (*p* < 0.001). Moreover, the clinical evidence associated with these variants also differed according to sex, with female patients harboring a higher percentage of molecular alterations targetable with approved drugs (*p* < 0.001) (Figure 4).

In this sense, smoking status also had an impact on the prevalence of actionable molecular alterations (*p* < 0.001). Eighty-seven percent of never-smoker patients presented harbored molecular alterations, with most of them (91%) being candidates for currently approved targeted therapies. Interestingly, no differences were observed in the percentage of patients with actionable molecular alterations or in their associated clinical evidence between former and current smokers. When comparing the frequency of actionable molecular alterations regarding sex and smoking status, female patients presented more targeted therapy options than men regardless of smoking status. In both female and male patients, no differences were observed between former and current smokers (Figure 4 and Figure S3).

Since NGS was established as the main molecular testing strategy, 128 samples had been prospectively analyzed using NGS. Of all these patients, 83 were diagnosed with de novo stage IV NSCLC, and 55 started systemic treatment for advanced disease. Based on the NGS results, 20 patients (36.4%) received at least one line of targeted treatment (Table 2). Moreover, among the remaining 35 patients, 8 (22.9%) could initiate targeted treatment after progression to their current treatment lines (6 with novel KRAS p.(Gly12Cys) inhibitors and two with novel EGFR exon 20 targeted agents).

### 3.5. First-Line Treatment Analyses

**Figure 5:**
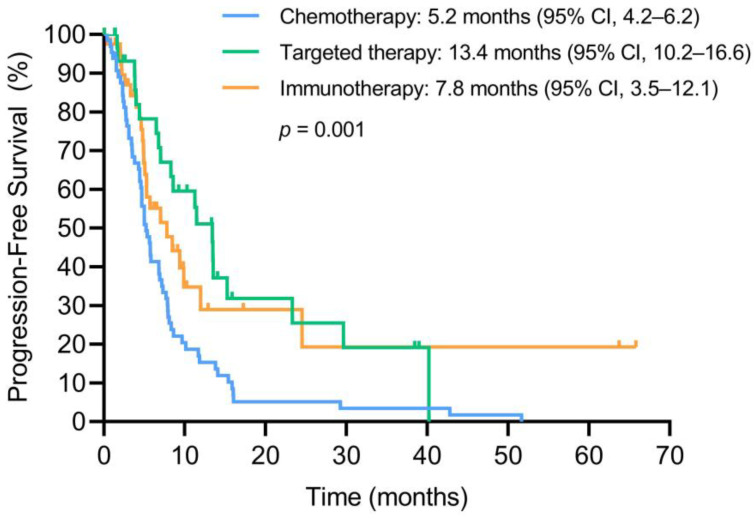
First-line progression-free survival of patients with stage IV NSCLC stratified by therapeutic approach.

The main clinical and pathological features of the 200 patients with de novo stage IV NSCLC included in the study are shown in Table 3. Among them, 146 started a first-line treatment for advanced disease: 69 (47.3%) were treated with chemotherapy, 33 (22.6%) were treated with targeted therapies, and 44 (30.1%) started an immunotherapy-based treatment regimen. The analysis of progression-free survival (PFS) showed statistically significant differences in the outcomes of patients according to each treatment strategy (*p* = 0.01). The patients treated with targeted therapy achieved a significantly longer PFS than the patients in the chemotherapy group: 13.4 months (95% CI, 10.2–16.6) vs. 5.2 months (95% CI, 4.2–6.2) (*p* = 0.001). In this comparison, chemotherapy treatment had a significantly higher risk of progression: HR: 2.3 (95% CI: 1.4–3.8). Similarly, the patients treated with immunotherapy achieved a longer median PFS (7.8 months (95% CI, 3.5–12.1)) (*p* = 0.011) and had a significantly higher risk of progression than those treated with chemotherapy (HR 1.8, (95% CI: 1.1–3.0)). Although the median PFS of the patients treated with targeted therapies was higher than that achieved by the patients treated with immunotherapies, these differences did not reach statistical significance. The outcomes of the immunotherapy-treated patients were diverse; while 31.6% of the patients progressed in the first 5 months after treatment began, 20.5% of the patients achieved prolonged responses (>60 months) (Figure 5).

### 3.6. Overall Survival

**Figure 6:**
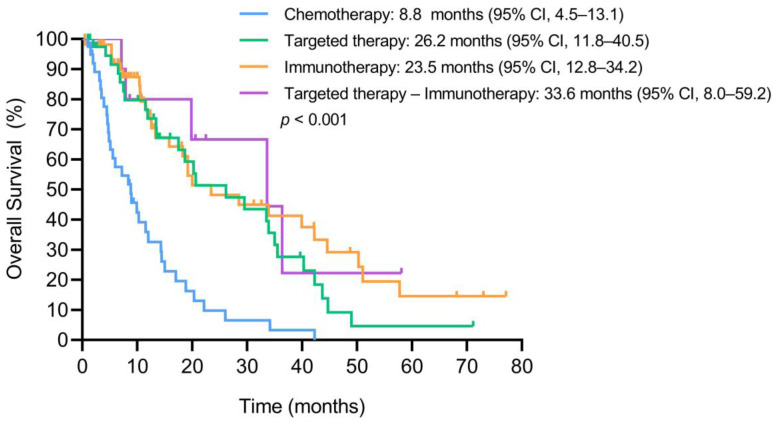
Overall survival of patients with stage IV NSCLC stratified by the treatment strategies administered during the course of the disease.

To evaluate the impact of the distinct treatment strategies on the overall survival (OS) of our patients with stage IV NSCLC (*n* = 200), we grouped them into three categories according to the treatment received: those who were treated only with chemotherapy (41, 28.1%), those who received at least one targeted therapy agent (40, 27.4%), those who initiated at least one immunotherapy-based regimen (55, 37.7%) and a subgroup of patients treated with both targeted therapies and immunotherapy (10, 6.9%). The overall survival was significantly different among the groups (*p* < 0.001), with the patients in the chemotherapy group showing the worst OS (8.8 months (95% CI, 4.5–13.1)) (Figure 6). Compared to the patients in the chemotherapy group, the patients treated with targeted therapy (HR: 0.3 (95% CI, 0.2–0.6)), immunotherapy (HR: 0.2 (95% CI, 0.1–0.4)) or both strategies (HR: 0.2 (95% CI, 0.1–0.6)) had a significantly decreased risk of death.

## 4. Discussion

Multiple targeted therapies are available for specific molecular subgroups of patients with NSCLC. The evolving landscape of this therapeutic approach requires molecular biology laboratories to implement the most up-to-date molecular techniques. In this scenario, NGS has rapidly positioned itself as the main molecular testing strategy for advanced NSCLC due to its ability to assess different molecular alterations with minimum sample requirements. However, the implementation of NGS into routine molecular diagnosis within the public healthcare system is still an unresolved issue, as recently emphasized by the Spanish Society of Pathology (SEAP) and the Spanish Society of Medical Oncology (SEOM) [15]. In this paper, we show the role of NGS certified by UNE-EN ISO 15189:2022 in understanding the molecular basis of NSCLC, as well as its impact on the clinical management of patients in our public healthcare hospital.

The clinical–pathological characteristics of the recruited patients show that our work is representative of the clinical reality of non-small-cell lung cancer in our community setting [16]. The frequency of molecular alterations in our cohort is concordant with that in previously published studies [17,18,19]. We also found significant differences in the molecular profile according to sex and smoking status. Sex has been considered a risk factor for lung cancer development, for instance, in women, lung cancer has been considered a separate disease with individual characteristics [20,21,22]. Moreover, distinct clinical outcomes have been reported between men and women, and the use of sex as a prognostic biomarker has also been discussed [23,24]. As previously reported, *EGFR* mutations and *ALK* fusion transcripts were found to be more frequent in female patients [25,26], while *TP53* mutations were significantly associated with male patients [27]. Regarding *ERBB2* mutations, our results are consistent with several articles suggesting their association with male sex [28,29].

The impact of smoking on the process of carcinogenesis indicates that NSCLC in non-smokers should be considered a separate disease with specific clinical features [30,31]. Our results support this observation, as the molecular profile of never-smoker patients was enriched in *EGFR* and *ERBB2* mutations, as well as in *ALK*, *ROS1*, *RET* and METEx14 fusion transcripts [32,33,34,35,36]. Interestingly, the heterogeneous group of former smoker patients may reflect the distinct evolution of NSCLC according to smoking history. In our cohort, a significantly lower smoking load was observed in former smokers with *EGFR* mutations, and, consequently they could be considered, NSCLC as “never smoker-like” patients [32].

The application of NGS to the diagnostics routine provided an overview of tumor heterogeneity, revealing co-occurring and mutual exclusion relationships among molecular alterations. In patients treated with targeted therapies, this information is particularly relevant since the strong selective pressure of treatment may enhance the proliferation of resistant clones [37,38]. *EGFR* activating mutations and *EGFR* gene amplification frequently co-occurred in our patients. This relationship has been previously reported, and in the majority of cases, amplification occurs in the mutated *EGFR* allele. Due to the higher amount of mutated *EGFR* in these tumors, these patients could constitute a unique subgroup in terms of responses to EGFR-TKI treatment [39,40]. As previously reported, in our cohort, *KRAS* mutations showed a mutual exclusion pattern with other major oncogenic drivers, such as *EGFR* mutations and *ALK* fusions [41,42]. In contrast, *KRAS* mutations were associated with *KRAS* amplifications, suggesting a synergistic role in driving cancer proliferation [43]. Interestingly, *KRAS* amplifications and *KRAS* amplifications were found to be concomitant in our cohort, which may reflect the cooperation of both alterations in NSCLC proliferation [44]. Finally, in our patients, an association relationship was identified between *MET* exon 14 skipping and *MET* gene amplification, which could be particularly relevant in terms of the response to *MET* inhibition treatment [45,46].

Sex- and smoking-related genetic differences in our cohort led to the distinct relevance of targeted therapies. The patients with actionable genetic alterations were most frequently female and never smokers [47]. Moreover, in these subgroups, actionable genetic alterations were mostly related to currently approved treatments. In this sense, in our cohort, never-smoker women constituted a unique subgroup of patients in which up to 91% could benefit from targeted treatment. Based on this percentage, maximum efforts should be made to offer a NGS study in this subgroup of patients [18,48]. In contrast, former smokers constitute a heterogeneous group in which smoking load and smoking abstinence should be taken into consideration. In our cohort, former and current smokers had a similar molecular profile. As previously reported, our results show a significantly lower prevalence of actionable alterations in tobacco-associated lung cancer [49].

In the patients with advanced NSCLC, PFS for first-line treatment was significantly different among therapeutic strategies. The chemotherapy-treated patients showed the worst outcome, while the targeted therapy-treated patients exhibited the longest PFS. Interestingly, the outcomes of the immunotherapy-treated patients were diverse; almost one-third of patients experienced disease progression in the first 5 months of treatment, while 20% of patients achieved prolonged responses. This result reflects the lack of robust biomarkers to identify patients who will benefit the most from this therapeutic approach [50,51].

The OS analysis of the patients included in this study clearly revealed the impact of targeted therapies and immunotherapies on the survival of patients with NSCLC. The patients who received at least one line of treatment based on this therapeutic approach experienced a significantly longer survival than those who only received chemotherapy-based regimens. These results justify the application of NGS to increase the number of patients harboring molecular alterations related to approved or ongoing clinical trials for targeted therapy [52,53,54].

Similarly to previously reported data in larger cohorts of real-life patients with NSCLC, we found a significantly better outcome of patients who received at least one line of targeted treatment or immunotherapy-based regimen [55,56].

Following the current recommendations, we integrated the NGS molecular profiling of advanced NSCLC into routine molecular diagnosis [10,11,12,13]. The accreditation of this testing strategy under the UNE-EN ISO 15189:2022 scope guarantees compliance with technical requirements, ensuring the reliability of the results and the consequent therapeutic decision making in a clinically practical turnaround time. The implementation of NGS has been a milestone for biomarker-selected treatments in our institution, since 36.4% of patients who started a first-line treatment have received targeted therapies based on the provided NGS results.

## 5. Conclusions

Our work shows the establishment of an NGS assay certified by UNE-EN ISO 15189:2022 in routine molecular diagnostics of NSCLC in a public reference healthcare hospital. Our results reveal distinct molecular profiles according to clinical–pathological features and provide insights into tumor heterogeneity. Moreover, targeted therapies were associated with better outcomes in our patients. The implementation of NGS has expanded the application of precision medicine to a greater number of patients.

## Figures and Tables

**Table 1:** Epidemiological and clinical–pathological characteristics of the recruited patients.

Variable	
**Age, mean ± SD**	63.2 ± 0.6
**Sex, *n* (%)**	
Male	217 (62.0)
Female	133 (38.0)
**Smoking history, *n* (%)**	
Never	65 (18.6)
Former smoker	127 (36.3)
Current smoker	158 (45.1)
**Smoking load (former and current smokers), median (IQR)**	36 (23–50)
**Years since quitting smoking (former smokers), median (IQR)**	12 (5–20)
**Histology, *n* (%)**	
Adenocarcinoma	288 (82.3)
Large-cell carcinoma	9 (2.6)
Squamous	14 (4.0)
Sarcomatoid carcinoma	11 (3.1)
Adenosquamous carcinoma	3 (0.9)
Large-cell neuroendocrine carcinoma	8 (2.3)
NOS	17 (4.9)
**Stage, *n* (%)**	
IA	38 (10.9)
IB	24 (6.9)
IIA	2 (0.6)
IIB	17 (4.9)
IIIA	28 (8.0)
IIIB	19 (5.4)
IIIC	9 (2.6)
IV	200 (57.1)
Unknown	13 (3.7)

SD: standard deviation. IQR: interquartile range.

**Table 2:** Patients with stage IV NSCLC treated with targeted therapies based on the NGS results.

Molecular Alteration	*n*	Drug
*EGFR*: p.(Leu858Arg)	4	Osimertinib
*EGFR*: p.(Glu746_Ala750del)	3	Osimertinib
*EGFR*: p.(Leu858Arg) + *EGFR* Amplification	1	Osimertinib
*EGFR*: p.(Gly719Ala) + p.(Ser768Ile)	1	Osimertinib
*EGFR*: p.(Leu861Gln)	1	Osimertinib
*EGFR*: p.(Glu709_Thr710delinsAsp)	1	Osimertinib
*EGFR*: p.(Ala767_Val769dup)	1	Amivantamab
EML4(13)-ALK(20)	1	Alectinib
KIF5B(17)-ALK(20)	1	Brigatinib
*ALK* Fusion (Unknown Partner)	1	Alectinib
MET(13)—MET(15)	1	Capmatinib
*KRAS*: p.(Gly12Cys)	1	Sotorasib
*BRAF*: p.(Val600Glu)	1	Dabrafenib + trametinib
SLC34A2(13)-ROS1(32)	1	Crizotinib
KIF5B(15)-RET(12) + *IDH1*: p.(Arg132His) + *MYC* Amplification	1	Selpercatinib

**Table 3:** Epidemiological and clinical–pathological characteristics of patients with stage IV NSCLC.

Variable	
**Age, mean ± SD**	63.1 ± 12.1
**Sex, *n* (%)**	
Male	78 (39.0)
Female	122 (61.0)
**Smoking history, *n* (%)**	
Never	46 (23.0)
Former smoker	63 (31.5)
Current smoker	91 (45.5)
**Sex and smoking history, *n* (%)**	
Never-smoker female	31 (15.5)
Former smoker female	16 (8.0)
Current smoker female	31 (15.5)
Never-smoker male	15 (7.5)
Former smoker male	47 (23.5)
Current smoker male	60 (30.0)
**Histology, *n* (%)**	
Adenocarcinoma	162 (81.0)
Large-cell carcinoma	5 (2.5)
Squamous	7 (3.5)
Sarcomatoid carcinoma	5 (2.5)
Adenosquamous carcinoma	2 (1.0)
Large-cell neuroendocrine carcinoma	5 (2.5)
NOS	14 (7.0)
**Systemic treatment, *n* (%)**	
No	37 (19.0)
Yes	158 (81.0)

SD: standard deviation.

## Data Availability

The data presented in this study are available upon reasonable request to the corresponding author.

## Supplementary Materials

The following supporting information can be downloaded at https://www.mdpi.com/article/10.3390/cancers15061705/s1, Figure S1: Smoking burden (pack/year) in former smokers according to the presence of activating mutations in the *EGFR* gene; Figure S2: Age at diagnosis according to the presence of METEx14 molecular alterations; Figure S3: Percentage of female and male patients harboring actionable genetic alterations according to smoking status.

## References

[1] Yang C.Y., Yang J.C.H., Yang P.C. (2020). Precision Management of Advanced Non-Small Cell Lung Cancer.

[2] Malone E.R., Oliva M., Sabatini P.J.B., Stockley T.L., Siu L.L. (2020). Molecular Profiling for Precision Cancer Therapies. Genome Med..

[3] Pennell N.A., Arcila M.E., Gandara D.R., West H. (2019). Biomarker Testing for Patients with Advanced Non-Small Cell Lung Cancer: Real-World Issues and Tough Choices. Am. Soc. Clin Oncol. Educ. Book.

[4] Arreaza G., Qiu P., Pang L., Albright A., Hong L.Z., Marton M.J., Levitan D. (2016). Pre-Analytical Considerations for Successful Next-Generation Sequencing (NGS): Challenges and Opportunities for Formalin-Fixed and Paraffin-Embedded Tumor Tissue (FFPE) Samples. Int. J. Mol. Sci..

[5] Isla D., Lozano M.D., Paz-Ares L., Salas C., de Castro J., Conde E., Felip E., Gómez-Román J., Garrido P., Enguita A.B. (2022). New Update to the Guidelines on Testing Predictive Biomarkers in Non-Small-Cell Lung Cancer: A National Consensus of the Spanish Society of Pathology and the Spanish Society of Medical Oncology. Clin. Transl. Oncol..

[6] Schneider F., Maurer C., Friedberg R.C. (2017). International Organization for Standardization (ISO) 15189. Ann. Lab. Med..

[7] ISO-ISO 15189:2012-Medical Laboratories—Requirements for Quality and Competence. https://www.iso.org/standard/56115.html.

[8] Smolle E., Pichler M. (2019). Non-Smoking-Associated Lung Cancer: A Distinct Entity in Terms of Tumor Biology, Patient Characteristics and Impact of Hereditary Cancer Predisposition. Cancers.

[9] Zhang T., Joubert P., Ansari-Pour N., Zhao W., Hoang P.H., Lokanga R., Moye A.L., Rosenbaum J., Gonzalez-Perez A., Martínez-Jiménez F. (2021). Genomic and Evolutionary Classification of Lung Cancer in Never Smokers. Nat. Genet..

[10] Garrido P., Conde E., de Castro J., Gómez-Román J.J., Felip E., Pijuan L., Isla D., Sanz J., Paz-Ares L., López-Ríos F. (2020). Updated Guidelines for Predictive Biomarker Testing in Advanced Non-Small-Cell Lung Cancer: A National Consensus of the Spanish Society of Pathology and the Spanish Society of Medical Oncology. Clin. Transl. Oncol..

[11] Mosele F., Remon J., Mateo J., Westphalen C.B., Barlesi F., Lolkema M.P., Normanno N., Scarpa A., Robson M., Meric-Bernstam F. (2020). Recommendations for the Use of Next-Generation Sequencing (NGS) for Patients with Metastatic Cancers: A Report from the ESMO Precision Medicine Working Group. Ann. Oncol..

[12] Penault-Llorca F., Kerr K.M., Garrido P., Thunnissen E., Dequeker E., Normanno N., Patton S.J., Fairley J., Kapp J., de Ridder D. (2022). Expert Opinion on NSCLC Small Specimen Biomarker Testing-Part 2: Analysis, Reporting, and Quality Assessment. Virchows Arch..

[13] Ettinger D.S., Wood D.E., Aisner D.L., Akerley W., Bauman J.R., Bharat A., Bruno D.S., Chang J.Y., Chirieac L.R., D’Amico T.A. (2022). Non-Small Cell Lung Cancer, Version 3.2022, NCCN Clinical Practice Guidelines in Oncology. J. Natl. Compr. Canc. Netw..

[14] Simarro J., Murria R., Pérez-Simó G., Llop M., Mancheño N., Ramos D., de Juan I., Barragán E., Laiz B., Cases E. (2019). Development, Implementation and Assessment of Molecular Diagnostics by next Generation Sequencing in Personalized Treatment of Cancer: Experience of a Public Reference Healthcare Hospital. Cancers.

[15] Jornada Medicina de Precisión | El Acceso a Determinaciones Moleculares Debe Estar Disponible En El SNS Para Aumentar La Supervivencia de Los Pacientes Con Cáncer | SEOM: Sociedad Española de Oncología Médica. https://seom.org/notas-prensa/209177-jornada-medicina-de-precision-el-acceso-a-determinaciones-moleculares-debe-estar-disponible-en-el-sns-para-aumentar-la-supervivencia-de-los-pacientes-con-cancer.

[16] Provencio M., Carcereny E., Rodríguez-Abreu D., López-Castro R., Guirado M., Camps C., Bosch-Barrera J., García-Campelo R., Ortega-Granados A.L., González-Larriba J.L. (2019). Lung Cancer in Spain: Information from the Thoracic Tumors Registry (TTR Study). Transl. Lung Cancer Res..

[17] Collisson E.A., Campbell J.D., Brooks A.N., Berger A.H., Lee W., Chmielecki J., Beer D.G., Cope L., Creighton C.J., Danilova L. (2014). Comprehensive Molecular Profiling of Lung Adenocarcinoma. Nature.

[18] Barlesi F., Mazieres J., Merlio J.P., Debieuvre D., Mosser J., Lena H., Ouafik L., Besse B., Rouquette I., Westeel V. (2016). Routine Molecular Profiling of Patients with Advanced Non-Small-Cell Lung Cancer: Results of a 1-Year Nationwide Programme of the French Cooperative Thoracic Intergroup (IFCT). Lancet.

[19] Tsoulos N., Papadopoulou E., Metaxa-Mariatou V., Tsaousis G., Efstathiadou C., Tounta G., Scapeti A., Bourkoula E., Zarogoulidis P., Pentheroudakis G. (2017). Tumor Molecular Profiling of NSCLC Patients Using next Generation Sequencing. Oncol. Rep..

[20] Isla D., Majem M., Viñolas N., Artal A., Blasco A., Felip E., Garrido P., Remón J., Baquedano M., Borrás J.M. (2017). A Consensus Statement on the Gender Perspective in Lung Cancer. Clin Transl. Oncol..

[21] Stapelfeld C., Dammann C., Maser E. (2020). Sex-Specificity in Lung Cancer Risk. Int. J. Cancer.

[22] Ragavan M., Patel M.I. (2022). The Evolving Landscape of Sex-Based Differences in Lung Cancer: A Distinct Disease in Women. Eur. Respir. Rev..

[23] Yu X.Q., Yap M.L., Cheng E.S., Ngo P.J., Vaneckova P., Karikios D., Canfell K., Weber M.F. (2022). Evaluating Prognostic Factors for Sex Differences in Lung Cancer Survival: Findings from a Large Australian Cohort. J. Thorac. Oncol..

[24] Ye Y., Jing Y., Li L., Mills G.B., Diao L., Liu H., Han L. (2020). Sex-Associated Molecular Differences for Cancer Immunotherapy. Nat. Commun..

[25] Zhang Y.L., Yuan J.Q., Wang K.F., Fu X.H., Han X.R., Threapleton D., Yang Z.Y., Mao C., Tang J.L. (2016). The Prevalence of EGFR Mutation in Patients with Non-Small Cell Lung Cancer: A Systematic Review and Meta-Analysis. Oncotarget.

[26] Fan L., Feng Y., Wan H., Shi G., Niu W. (2014). Clinicopathological and Demographical Characteristics of Non-Small Cell Lung Cancer Patients with ALK Rearrangements: A Systematic Review and Meta-Analysis. PLoS ONE.

[27] Haupt S., Caramia F., Herschtal A., Soussi T., Lozano G., Chen H., Liang H., Speed T.P., Haupt Y. (2019). Identification of Cancer Sex-Disparity in the Functional Integrity of P53 and Its X Chromosome Network. Nat. Commun..

[28] Arcila M.E., Chaft J.E., Nafa K., Roy-Chowdhuri S., Lau C., Zaidinski M., Paik P.K., Zakowski M.F., Kris M.G., Ladanyi M. (2012). Prevalence, Clinicopathologic Associations, and Molecular Spectrum of ERBB2 (HER2) Tyrosine Kinase Mutations in Lung Adenocarcinomas. Clin. Cancer Res..

[29] Bu S., Wang R., Pan Y., Yu S., Shen X., Li Y., Sun Y., Chen H. (2017). Clinicopathologic Characteristics of Patients with HER2 Insertions in Non-Small Cell Lung Cancer. Ann. Surg. Oncol..

[30] Yu X., Chen G., Yang J., Yu G., Zhu P., Jiang Z., Feng K., Lu Y., Bao B., Zhong F. (2019). Smoking Alters the Evolutionary Trajectory of Non-Small Cell Lung Cancer. Exp. Ther. Med..

[31] Li X., Huang C., Xie X., Wu Z., Tian X., Wu Y., Du X., Shi L. (2021). The Impact of Smoking Status on the Progression-Free Survival of Non-Small Cell Lung Cancer Patients Receiving Molecularly Target Therapy or Immunotherapy versus Chemotherapy: A Meta-Analysis. J. Clin. Pharm. Ther..

[32] Chapman A.M., Sun K.Y., Ruestow P., Cowan D.M., Madl A.K. (2016). Lung Cancer Mutation Profile of EGFR, ALK, and KRAS: Meta-Analysis and Comparison of Never and Ever Smokers. Lung Cancer.

[33] Dias M., Linhas R., Campainha S., Conde S., Barroso A. (2017). Lung Cancer in Never-Smokers-What Are the Differences?. Acta Oncol..

[34] Wei X.W., Gao X., Zhang X.C., Yang J.J., Chen Z.H., Wu Y.L., Zhou Q. (2020). Mutational Landscape and Characteristics of ERBB2 in Non-Small Cell Lung Cancer. Thorac. Cancer.

[35] Zhu Q., Zhan P., Zhang X., Lv T., Song Y. (2015). Clinicopathologic Characteristics of Patients with ROS1 Fusion Gene in Non-Small Cell Lung Cancer: A Meta-Analysis. Transl. Lung Cancer Res..

[36] Wang R., Hu H., Pan Y., Li Y., Ye T., Li C., Luo X., Wang L., Li H., Zhang Y. (2012). RET Fusions Define a Unique Molecular and Clinicopathologic Subtype of Non-Small-Cell Lung Cancer. J. Clin. Oncol..

[37] Seoane J., de Mattos-Arruda L. (2014). The Challenge of Intratumour Heterogeneity in Precision Medicine. J. Intern. Med..

[38] Zhang J., Späth S.S., Marjani S.L., Zhang W., Pan X. (2018). Characterization of Cancer Genomic Heterogeneity by Next-Generation Sequencing Advances Precision Medicine in Cancer Treatment. Precis. Clin. Med..

[39] Sholl L.M., Yeap B.Y., Iafrate A.J., Holmes-Tisch A.J., Chou Y.P., Wu M.T., Goan Y.G., Su L., Benedettini E., Yu J. (2009). Lung Adenocarcinoma with EGFR Amplification Has Distinct Clinicopathologic and Molecular Features in Never-Smokers. Cancer Res..

[40] Ruiz-Patiño A., Castro C.D., Ricaurte L.M., Cardona A.F., Rojas L., Zatarain-Barrón Z.L., Wills B., Reguart N., Carranza H., Vargas C. (2018). EGFR Amplification and Sensitizing Mutations Correlate with Survival in Lung Adenocarcinoma Patients Treated with Erlotinib (MutP-CLICaP). Target. Oncol..

[41] Gainor J.F., Varghese A.M., Ou S.H.I., Kabraji S., Awad M.M., Katayama R., Pawlak A., Mino-Kenudson M., Yeap B.Y., Riely G.J. (2013). ALK Rearrangements Are Mutually Exclusive with Mutations in EGFR or KRAS: An Analysis of 1,683 Patients with Non-Small Cell Lung Cancer. Clin. Cancer Res..

[42] Timar J., Kashofer K. (2020). Molecular Epidemiology and Diagnostics of KRAS Mutations in Human Cancer. Cancer Metastasis Rev..

[43] Wagner P.L., Stiedl A.C., Wilbertz T., Petersen K., Scheble V., Menon R., Reischl M., Mikut R., Rubin M.A., Fend F. (2011). Frequency and Clinicopathologic Correlates of KRAS Amplification in Non-Small Cell Lung Carcinoma. Lung Cancer.

[44] Goh K.Y., Lim W.T. (2020). Cyclin D1 Expression in KRAS Mutant Non-Small Cell Lung Cancer-Old Wine into New Skins. Transl. Lung Cancer Res..

[45] Awad M.M., Oxnard G.R., Jackman D.M., Savukoski D.O., Hall D., Shivdasani P., Heng J.C., Dahlberg S.E., Jänne P.A., Verma S. (2016). MET Exon 14 Mutations in Non-Small-Cell Lung Cancer Are Associated with Advanced Age and Stage-Dependent MET Genomic Amplification and c-Met Overexpression. J. Clin Oncol..

[46] Tong J.H., Yeung S.F., Chan A.W.H., Chung L.Y., Chau S.L., Lung R.W.M., Tong C.Y., Chow C., Tin E.K.Y., Yu Y.H. (2016). MET Amplification and Exon 14 Splice Site Mutation Define Unique Molecular Subgroups of Non-Small Cell Lung Carcinoma with Poor Prognosis. Clin. Cancer Res..

[47] Devarakonda S., Li Y., Rodrigues F.M., Sankararaman S., Kadara H., Goparaju C., Lanc I., Pepin K., Waqar S.N., Morgensztern D. (2021). Genomic Profiling of Lung Adenocarcinoma in Never-Smokers. J. Clin. Oncol..

[48] Sholl L.M., Aisner D.L., Varella-Garcia M., Berry L.D., Dias-Santagata D., Wistuba I.I., Chen H., Fujimoto J., Kugler K., Franklin W.A. (2015). Multi-Institutional Oncogenic Driver Mutation Analysis in Lung Adenocarcinoma: The Lung Cancer Mutation Consortium Experience. J. Thorac. Oncol..

[49] Löfling L., Karimi A., Sandin F., Bahmanyar S., Kieler H., Lambe M., Lamberg K., Wagenius G. (2019). Clinical Characteristics and Survival in Non-Small Cell Lung Cancer Patients by Smoking History: A Population-Based Cohort Study. Acta Oncol..

[50] Bylicki O., Barazzutti H., Paleiron N., Margery J., Assié J.B., Chouaïd C. (2019). First-Line Treatment of Non-Small-Cell Lung Cancer (NSCLC) with Immune Checkpoint Inhibitors. BioDrugs.

[51] Proto C., Ferrara R., Signorelli D., lo Russo G., Galli G., Imbimbo M., Prelaj A., Zilembo N., Ganzinelli M., Pallavicini L.M. (2019). Choosing Wisely First Line Immunotherapy in Non-Small Cell Lung Cancer (NSCLC): What to Add and What to Leave Out. Cancer Treat. Rev..

[52] Tsimberidou A.-M., Hong D.S., Ye Y., Cartwright C., Wheler J.J., Falchook G.S., Naing A., Fu S., Piha-Paul S., Janku F. (2017). Initiative for Molecular Profiling and Advanced Cancer Therapy (IMPACT): An MD Anderson Precision Medicine Study. JCO Precis. Oncol..

[53] Schwaederle M., Zhao M., Lee J.J., Eggermont A.M., Schilsky R.L., Mendelsohn J., Lazar V., Kurzrock R. (2015). Impact of Precision Medicine in Diverse Cancers: A Meta-Analysis of Phase II Clinical Trials. J. Clin. Oncol..

[54] Pakkala S., Ramalingam S.S. (2018). Personalized Therapy for Lung Cancer: Striking a Moving Target. JCI Insight.

[55] Lester J., Escriu C., Khan S., Hudson E., Mansy T., Conn A., Chan S., Powell C., Brock J., Conibear J. (2021). Retrospective Analysis of Real-World Treatment Patterns and Clinical Outcomes in Patients with Advanced Non-Small Cell Lung Cancer Starting First-Line Systemic Therapy in the United Kingdom. BMC Cancer.

[56] Shokoohi A., Al-Hashami Z., Moore S., Pender A., Wong S.K., Wang Y., Leung B., Wu J., Ho C. (2022). Effect of Targeted Therapy and Immunotherapy on Advanced Nonsmall-Cell Lung Cancer Outcomes in the Real World. Cancer Med..

