# Supplementary figures and images for: Impact of Molecular Testing Using Next-Generation Sequencing in the Clinical Management of Patients with Non-Small Cell Lung Cancer in a Public Healthcare Hospital
Source: Cancers (Basel). 2023 Mar 10;15(6):1705. doi: 10.3390/cancers15061705 (PMC10046107; doi:10.3390/cancers15061705)

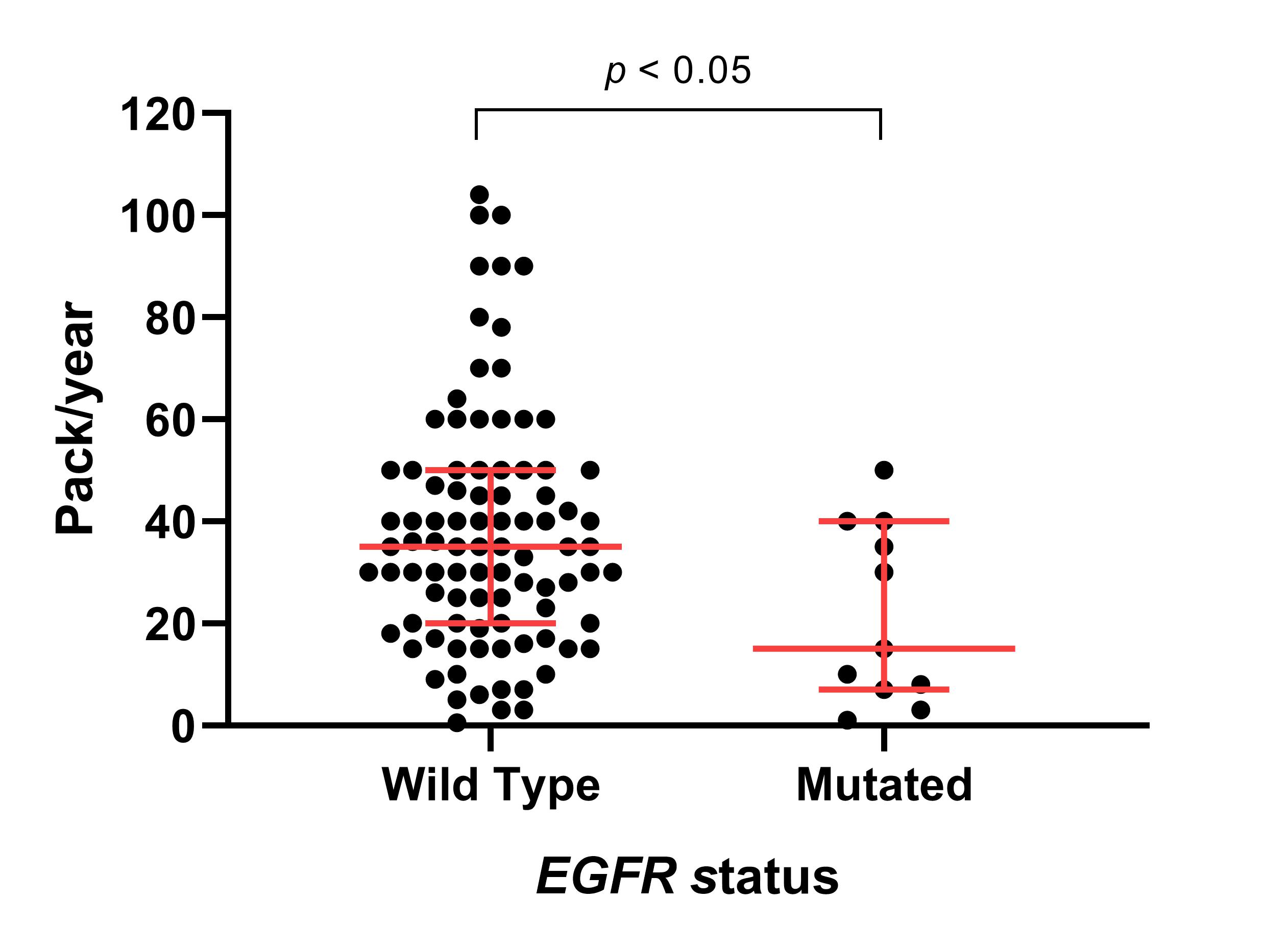

Supplement: Supplementary file 1 [file cancers-15-01705-s001.zip › Figure S1. Smoking burden EGFR.jpg]

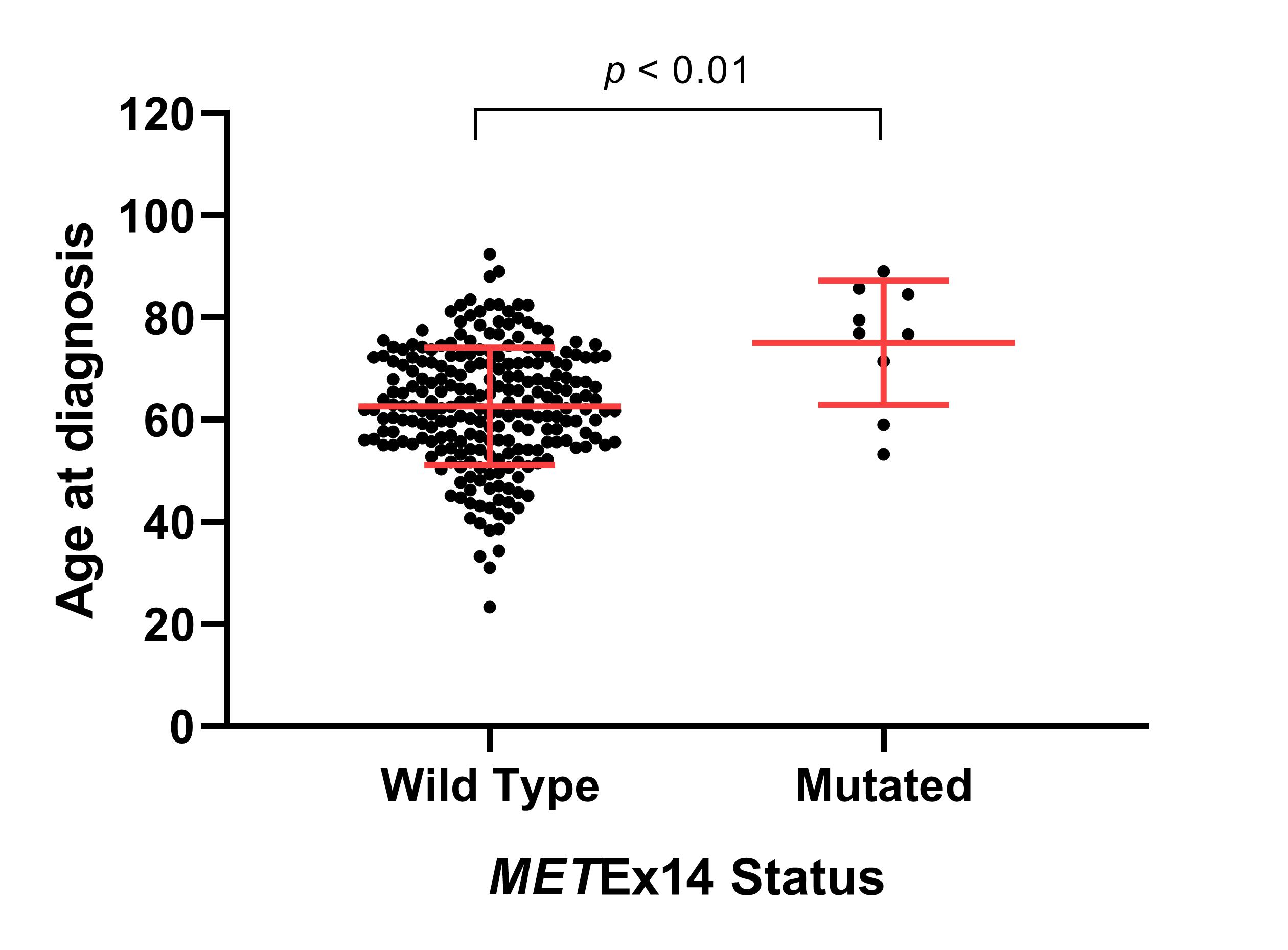

Supplement: Supplementary file 1 [file cancers-15-01705-s001.zip › Figure S2. Age at diagnosis METEx14.jpg]

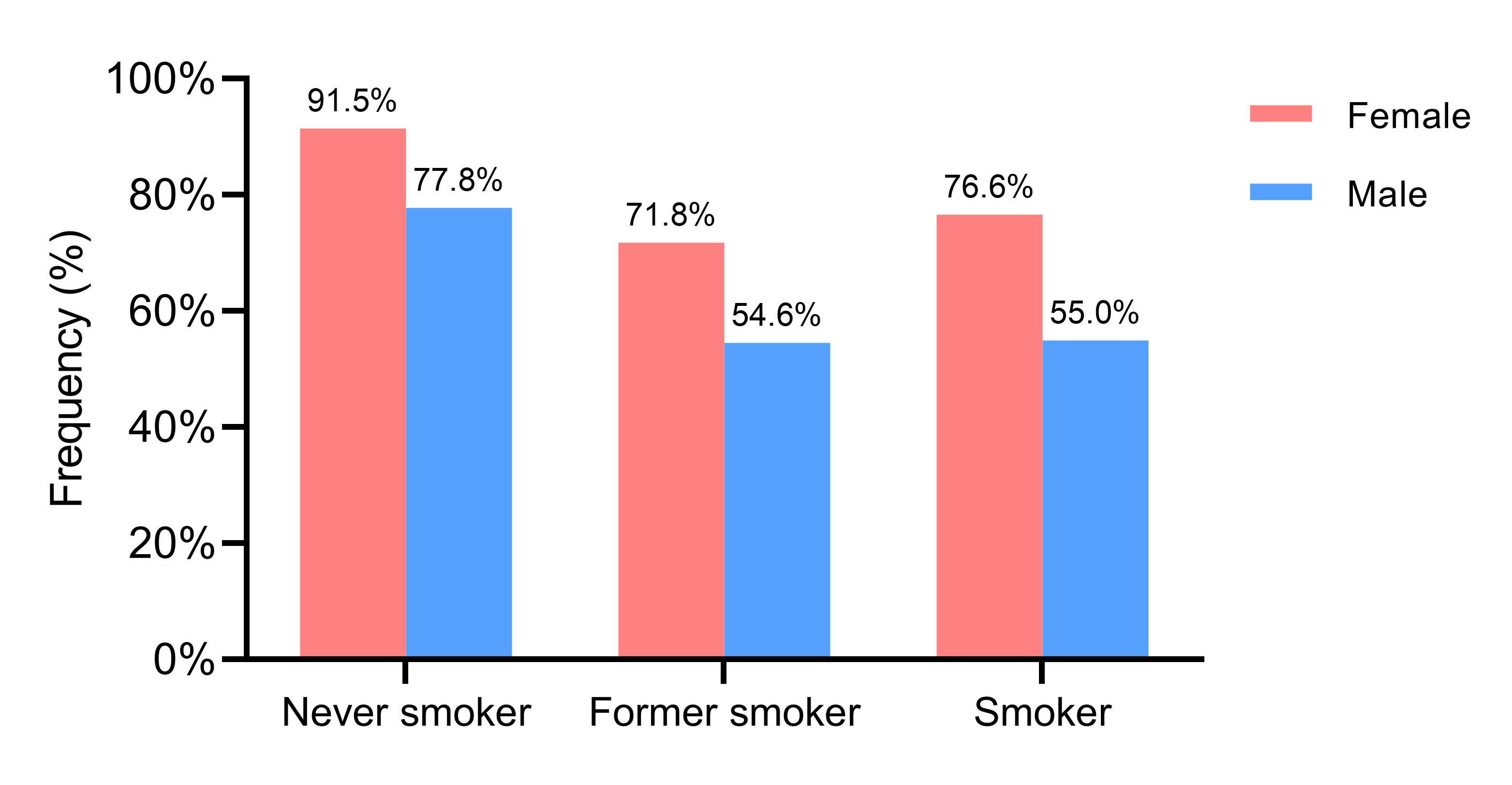

Supplement: Supplementary file 1 [file cancers-15-01705-s001.zip › Figure S3. Percentage of female and male patients.jpg]
